# ‘It's not what you'd term normal smoking’: a qualitative exploration of language used to describe heated tobacco product use and associated user identity

**DOI:** 10.1111/add.16051

**Published:** 2022-10-11

**Authors:** Katherine A. East, Connor R. Miller, Sara C. Hitchman, Ann McNeill, Charlotte N. E. Tompkins

**Affiliations:** ^1^ National Addiction Centre, Department of Addictions, Institute of Psychiatry, Psychology & Neuroscience King's College London London UK; ^2^ Department of Health Behavior Roswell Park Comprehensive Cancer Center Buffalo New York USA; ^3^ Department of Communication and Media Research University of Zurich Zurich Switzerland

**Keywords:** Heated tobacco, identity, language, qualitative research, smoking, tobacco use

## Abstract

**Background and aims:**

Tobacco and nicotine marketplaces have diversified over the past decade, including with the introduction of heated tobacco products (HTPs), such as the brand IQOS. HTPs typically heat tobacco to generate an aerosol that is inhaled. HTP nomenclature is lacking, and how HTP users define and identify themselves remains understudied. Research in this area is important because language can construct identity, and identity can shape behaviour. This study aimed to explore users' language choice when describing IQOS use, and how language relates to user identity.

**Methods:**

Qualitative interviews in London, United Kingdom, with 30 adult current and former IQOS users. Analyses were guided by Iterative Categorization.

**Results:**

Overall, participants expressed confusion and a lack of suitable terminology for how to describe IQOS use. Verbs such as *heating* and *IQOSing* were rarely endorsed. Most often, participants reverted to *smoking* when describing IQOS use and commonly referred to HEETS (tobacco sticks) as *cigarettes*. Yet the lack of combustion, electronic device, *cleaner* experience and perceived reductions in health risks led some to frame IQOS as distinct from smoking. *Vaping* was generally considered inappropriate for describing IQOS use. Participants also manipulated language to suit their circumstances and manage their identity, whereas some IQOS users embraced the terms *smoking* and *smoker*, most were eager to distinguish between using IQOS and being labelled *a smoker* because of the associated negative connotations and to align with perceptions of IQOS use as a *better*, less harmful behaviour. Instead, when describing their identity, IQOS users more willingly identified as *vapers*, or *ex‐smokers*, or created new identities (e.g. *HEET user*).

**Conclusions:**

People who use or have used IQOS (a brand of heated tobacco product) are ambiguous about IQOS terminology. Participants in this study commonly referred to IQOS use as *smoking* for lack of a more suitable term, but also resisted being labelled as *smokers*, a choice that may influence smoking cessation. Clear terminology must be used in surveys and by healthcare professionals when asking about cigarette smoking and e‐cigarette and heated tobacco product use.

## INTRODUCTION

The tobacco and nicotine marketplace has experienced unprecedented diversification over the past decade [1], including the introduction of heated tobacco products (HTPs) such as glo, Ploom and IQOS [2]. HTPs are electronic devices manufactured by tobacco companies, such as Philip Morris International. HTPs typically heat tobacco to generate an aerosol, and are hence purported to be less harmful than smoking combustible cigarettes. IQOS is especially widespread, being commercialised in 64 markets as of 2020 [3]. In the United Kingdom (UK), IQOS has been marketed since 2016, although current use remains low at <1% and almost entirely among current or former smokers [4].

Precise and consistent nomenclature is lacking for HTPs [2, 5]. Many HTPs, including IQOS, use tobacco sticks that resemble combustible cigarettes in shape and branding [2]. Conversely, the electronic nature of HTP devices resemble e‐cigarettes [2]. Tobacco companies have marketed HTPs as ‘heat‐not‐burn’ products, ‘tobacco vapour products’, ‘heated tobacco systems’ and ‘tobacco heating products’ [2, 5].

Language can construct identity, which in turn can shape behaviour [6, 7]. For example, smokers who quit but continue to identify as a smoker are at greater risk of relapse [8], whereas those who identify as a non‐smoker are more likely to stay quit [9, 10]. Some smokers also reject vaping because vaping does not fit within their smoker identity [11]; identity could, therefore, hinder switching to a different nicotine product. It is unclear how HTP users define and identify themselves and how this impacts HTP use and smoking. Understanding HTP user identity and terminology can also inform survey design such as how to refer to HTPs and HTP use.

We, therefore, qualitatively explore the language used to describe use of the leading HTP in the United Kingdom (IQOS) and consider how language relates to user identity.

## METHODS

Methods are described in the Appendix S1 and previous publications [12, 13]. Briefly, 30 UK residents ages 18+ years that currently/formerly used IQOS and smoked combustible cigarettes were interviewed between 2018 and 2019 about their experiences using IQOS. In the first interviews, participants (often unprompted) raised thoughts about how to refer to using IQOS and engaged in detailed discussions about appropriate terminology. Use of language was probed in all subsequent interviews.

Analyses were guided by Iterative Categorization [14, 15], a systematic and staged approach to qualitative data analyses. For this paper, we systematically reviewed, inductively consolidated, and re‐organised data within the ‘language’ and ‘identity’ codes [15]. This included exploring the data for differences by age, gender and smoking and vaping experiences, and considering findings within the broader context and established knowledge.

## FINDINGS

Table 1 shows the sample characteristics. Most currently used IQOS. While all but two had tried vaping e‐cigarettes, most did not currently vape (*n* = 26) and/or had unsatisfactory experiences with vaping.

**TABLE 1:** Sample characteristics (*n* = 30)

*Characteristics*	*Participants in sample (n)*
Age (years)	
18–24	7
25–34	8
35–49	12
50–59	3
Gender	
Male	19
Female	11
Ethnicity	
White British	8
White Other	17
Asian British	3
Black British	1
Arabic	1
Occupation	
Professional/qualified	9
Managerial/senior administrator	9
Clerical/junior administrator	8
Sales/services	1
Semi‐skilled/unskilled labour	1
Never worked	2
Patterns of current IQOS use and smoking	
Current IQOS user	22
(current smoking: daily/weekly/monthly/less than monthly)	(15)
(current smoking: not at all)	(7)
Former IQOS user	8
(current smoking: daily/weekly/monthly/less than monthly)	(6)
(current smoking: not at all)	(2)
Length of IQOS use	
1–3 months	4
4–6 months	7
7–12 months	11
More than 12 months	8
Current frequency of cigarette smoking	
Daily	7
Weekly	4
Monthly	4
Less than monthly	6
Not at all	9
Time using tobacco and nicotine products^a^	
1–5 years	9
6–10 years	5
11–20 years	3
More than 20 years	12

^a^ Includes use of other tobacco and nicotine products (e.g. shisha and cigars). One participant did not disclose time since using tobacco and nicotine products, so missing data exist.

### A ‘new way’ of smoking

#### Lack of suitable terminology

Overall, when discussing what language best describes the act of using IQOS, participants expressed confusion and reported a lack of suitable terminology. Participants commonly used the brand name *IQOS* when referencing the device, but tended to shy away from using this when describing use because it sounded *pretentious*, *gimmicky*, or was not widely recognised. *IQOSing* was not considered a useable verb. Similarly, *heat‐not‐burn* or *heating‐not‐burning* were considered unsuitable because of their long‐winded nature, and the verb *heating* was deemed inappropriate given its alternative contextual use as a source of warmth.

I'm not really sure what to call it … IQOS is not the best term … it's a bit hard to say, IQOS, and you certainly could never say IQOSing … that's way too much of a tongue twister! … It just doesn't trip off your tongue very easily … HEETing sounds a bit weird. (Raj, age 43)

### Appraising alternative terminology

Participants, therefore, generally understood their own use of IQOS as *smoking* and used phrases such as *going to smoke* when describing their behaviour to others. This was based on their perceptions that the physical act and sensation of using IQOS closely *mimics* smoking combustible cigarettes: it has the *same tobacco taste*, provides a similar *throat hit*, involves the same *rituals*, has a defined start and end point and produces a smoke‐like exhale, albeit less voluminous. Furthermore, *smoking* was perceived to be a more understandable reference for others, partly out of habit from prior use of combustible cigarettes, but also because of the lack of suitable alternative language to describe IQOS use, and because it was considered a more all‐encompassing term across different tobacco and nicotine use behaviours that involve inhalation.

Somebody asks me if I smoke, I say that yes, I smoke … I describe it [IQOS] as smoking, because it's a tobacco product and it's similar to smoking. It also means less having to explain what it is! (Luca, age 24)

Furthermore, participants commonly referred to HEETS as *cigarettes* (including *little cigarette* or *IQOS cigarette*) and explained that, like combustible cigarettes, HEETS contained tobacco, were sold in packets of 20, and *butts* were discarded after use. Considering these similarities, some described using IQOS as *going for a cigarette* and considered that *puffing* and *dragging* accurately described IQOS use.

Despite the similarities, the term *smoking* was sometimes used with an acknowledgement of the limits to which using IQOS fully mimics the experience of smoking combustible cigarettes. That is, the lack of burning/combustion, the electronic device, and *cleaner* and *lighter* experiences of use, alongside perceived reductions in health risks [13], were noted as key differences from *normal smoking*. These differences led some participants to perceive IQOS as *smoking for the 21st century*, aligning with a shift toward a more *tech savvy* and *futuristic* way of smoking.

Participants also drew comparisons with vaping e‐cigarettes. Similarities included the electronic device, lack of combustion, *vapour* or *steam*‐like emissions and perceptions about reduced harms. Nevertheless, some (particularly daily smokers and those with negative experiences of e‐cigarettes) keenly positioned IQOS use as different to vaping because of the more discrete device, use of tobacco‐containing HEETS (rather than e‐liquid), sensory differences, no *cloud* of emissions, and more distinct duration of use. Reflecting this, *vaping* was not widely considered an appropriate verb to describe IQOS use.

I would definitely not call it either smoking or vaping, because … I associate vaping with e‐liquid … smoke comes from burning, and this one doesn't burn, so it's like not smoking … there's no verb to describe doing IQOS, apart from doing. (Yulia, age 19)

Overall, appraising these similarities and differences led participants to position IQOS *between* smoking combustible cigarettes and vaping e‐cigarettes or as a *hybrid* or *halfway house* of both behaviours, despite the overall closer experience, satisfaction and *ritual* of using IQOS to smoking than vaping.

I can only compare it to smoking or vaping … on that spectrum, it's nearer to smoking than vaping … vaping is common enough now that … if you said … ‘I'm vaping’, people would understand that in a certain way, and I don't think IQOS is the same. (Sanjay, age 43)

### Impact on identity

Participants' language about using IQOS reflected their perceived impacts on image and identity. On account of still using a tobacco product in much the same way as combustible cigarettes, IQOS users (including those who stopped smoking combustibles) often identified as *smokers*.

I feel that I'm still a smoker, because I need to do the same actions if I want to smoke. (Ana, age 30)

Nevertheless, participants were sometimes conflicted in using the term *smoker* when describing their identity in relation to IQOS. Many were eager to distinguish between using IQOS and being *a smoker* because of the prevailing *dirty* connotations and *social taboo* of being a combustible cigarette smoker. Participants also generally perceived that using IQOS positively impacted personal image because it symbolised an attempt to be more *health conscious* through adopting a less *risky*, more *responsible* behaviour.

I don't know how to define myself anymore … I use HEETS, but I don't burn cigarettes … I guess I use tobacco products … Now I use HEETS, am I a smoker? I don't know. (Sean, age 52)

To help manage their perceived shift in behaviour and identity, participants manipulated their language to suit their circumstances. Those keen to dissociate themselves from combustible cigarettes sometimes intentionally distinguished using IQOS as having made an *absolute conscious decision* to stop smoking and avoid being *vilified* and labelled as a smoker. This involved creating new identities, such as *HEET user*, to underscore their *deliberate choice* to switch, whereas others viewed themselves as *ex‐smokers* who had used IQOS to *stop smoking* or as an *alternative* to, or *substitute* for, smoking. This led some current IQOS users, including those who perceived using IQOS as a form of smoking, to question whether they would be considered *a smoker* on medical *forms* and for life and travel *insurance* purposes.

[A medical form] asked whether I smoked cigarettes. I didn't actually know how to categorise it … it even gave an option for, ‘do you vape’ … But I wouldn't actually know, so I kind of left an additional comment describing that I'm having IQOS and it steams it as opposed to burning … that was kind of the confusion when filling out the form. (Yusuf, age 25)

Women in particular felt that using IQOS looked *elegant*, *artistic* and *sophisticated*. Yet, two female students in their 20s vocalised concerns about their identity among *basic cigarette* smokers. They experienced *privileged guilt* and felt like *fake cigarette smokers* who no longer fit in with the *smoking community* on account of the *less shared experience* by using *a hipster device* [IQOS].

Only one participant considered the term *vaper* accurate and acceptable to define their identity. However, in places where smoking was prohibited and when talking to family with anti‐smoking opinions, participants more willingly used e‐cigarette‐related language and identified as *vapers* to *conveniently fit* their *narrative* or align with their family's perception of a *better* behaviour, despite believing themselves that using IQOS was not *vaping* per se.

IQOS is to them [children], it's a form of vaping, which is acceptable … ‘I'm going for an IQOS’, that's what I might say to my wife … to the children I might say, ‘I'm going for a vape’. To me it's like it's a smoke … there is a perception of smokers as bad these days … it's not nearly so bad if it's seen to be a vape or a smoking alternative. (William, age 36)

## DISCUSSION

This paper explored language used to describe IQOS and its relationship to user identity. Overall, there was great ambiguity around verb terminology. Consistent with prior research on vaping [11], IQOS users generally articulated their IQOS use and identity in relation to existing smoking identities; specifically, participants referred to IQOS use as ‘smoking’ for lack of a more suitable and commonly understood term. Yet, users were eager to distinguish themselves from being labelled as ‘smokers’ because of the associated negative connotations.

Findings have important implications for tobacco research and clinical practice. First, we found that language can help construct and manage identity relating to IQOS use and smoking. Prior research suggests smoker identity can impact smoking abstinence [6, 7, 8, 9, 10] and trying less harmful products such as e‐cigarettes [11]. Our finding that IQOS users are generally eager to dissociate from being ‘a smoker’ may, therefore, be promising for maintaining smoking abstinence; however, the fluidity of identity between ‘IQOS user’ and ‘smoker’ could increase concurrent use. Second, consistent with ‘Addiction Ontology’ [16], researchers should use clear terminology when asking people to report their smoking and other nicotine/tobacco product use by providing specific examples of products (e.g. combustible tobacco cigarettes), including brands or photographs where appropriate, to avoid confusion and enhance validity. As ‘heat‐not‐burn’ and ‘heating’ were not considered appropriate, such terminology could be avoided. Third, healthcare professionals also should use appropriate terminology when asking patients about their nicotine/tobacco use, to ensure that the products used are correctly assessed and provide optimum advice and treatment options. Finally, findings can contribute toward nicotine/tobacco ontologies and meta‐syntheses of HTP research [16].

Limitations include that our sample comprised adults <60 years old who were mainly in professional occupations, unlike typical combustible cigarette smokers in the United Kingdom [13], so our findings may not generalise beyond these groups. Strengths include using multiple recruitment methods to access a sample with diverse experiences and one‐to‐one in‐depth interviews to have detailed conversations on IQOS terminology and identity.

## DECLARATION OF INTERESTS

A.M. is a National Institute for Health Research (NIHR) Senior Investigator. The views expressed in this article are those of the authors and not necessarily those of the NIHR or the Department of Health and Social Care. All other authors have no conflict of interest to declare.

## AUTHOR CONTRIBUTIONS

**Katherine A. East:** Conceptualization; formal analysis; funding acquisition; investigation. **C** **onnor R. Miller:** Investigation. **Sara C. Hitchman:** Conceptualization; data curation; funding acquisition; investigation; methodology; project administration; resources; supervision. **Ann McNeill:** Conceptualization; funding acquisition; investigation; methodology; project administration; resources; supervision. **Charlotte N. E. Tompkins:** Conceptualization; data curation; formal analysis; funding acquisition; investigation; methodology; project administration; supervision.

## Supporting information


**Appendix S1** ‘It's not what you'd term normal smoking’: A qualitative exploration of language used to describe heated tobacco product use and associated user identity
