# Supplementary material for: ‘It's not what you'd term normal smoking’: a qualitative exploration of language used to describe heated tobacco product use and associated user identity
Source: Addiction. 2022 Oct 11;118(3):533–8. doi: 10.1111/add.16051 (PMC9898082; doi:10.1111/add.16051)
Supplement: Supplementary file 1 — Appendix S1 ‘It's not what you'd term normal smoking’: A qualitative exploration of language used to describe heated tobacco product use and associated user identity [file ADD-118-533-s001.docx]

**APPENDIX: ‘It’s not what you’d term normal smoking’: A qualitative exploration of language used to describe Heated Tobacco Product (HTP) use and associated user identity**

Eligibility criteria were adults (18+) who (1) currently smoked or quit smoking in the last 2 years, (2) use or used IQOS at least weekly for at least 1 month, and (3) lived in the UK. We recruited online (research recruitment website, classifieds, social media) and via a university-wide email. Posters were also displayed in vape shops selling IQOS across London and we approached individuals using IQOS in public. After screening, eligible individuals were selected for interview based on their demographics and histories of smoking and IQOS use.

Between October 2018 and February 2019, CNET, qualitatively interviewed 30 people. A semi structured topic guide, informed by consultations with seven tobacco/nicotine experts and a panel of 12 current and ex-smokers, guided the interviews. Interviews used an open and flexible questioning style and aimed to explore participants’ experiences using IQOS. In the first interviews, participants (often unprompted) raised thoughts about how to refer to using IQOS and engaged in detailed discussions about the appropriateness of different terminology. Use of language was probed in all subsequent interviews to ascertain participants’ views. Interviews lasted 36–102 min (mean=67).

Analyses were guided by Iterative categorisation, a systematic and staged approach to qualitative data management and analyses. The authors read transcripts to familiarise themselves with the data, discussed the content, and developed a coding frame. The transcripts and the coding frame were imported into MaxQDA; each transcript was systematically coded whereby all text was assigned to the relevant code/s. Then, we systematically reviewed, inductively consolidated, and re-organised data within the ‘language’ and ‘identity’ codes during the interpretative analytical stages. This included exploring the data for differences by age, gender, and smoking and vaping experiences, and considering findings within the broader context and established knowledge. Key findings are illustrated using pseudonymised verbatim quotations.
